# Genetic variant reanalysis reveals a case of Sandhoff disease with onset of infantile epileptic spasm syndrome

**DOI:** 10.1186/s42494-024-00149-4

**Published:** 2024-03-08

**Authors:** Qi Zhang, Liping Zou, Qian Lu, Qiuhong Wang, Shuo Dun, Jing Wang

**Affiliations:** 1 Medical School of Chinese PLA, Beijing, 100853 China; 2 Department of Pediatrics, the First Medical Center of PLA General Hospital, Beijing, 100853 China

**Keywords:** Infantile Sandhoff disease, Gene variant reanalysis, *HEXB* gene, Infantile epilepsy spasm syndrome, Cherry red spot, Human phenotype ontology

## Abstract

**Background:**

Sandhoff disease (SD) i s an autosomal recessive lysosomal disease with clinical manifestations such as epilepsy, psychomotor retardation and developmental delay. However, infantile SD with onset of infantile epilepsy spasm syndrome (IESS) is extremely rare.

**Case presentation:**

The case presented here was a 22-month-old boy, who presented with IESS and psychomotor retardation/regression at 6 months of age. The patient showed progressive aggravation of seizures and excessive startle responses. The whole exome sequencing data, which initially revealed negative results, were reanalyzed and indicated a homozygous mutation at the c.1613 + 4del splice site of the *HEXB* gene. The activities of β-hexosaminidase A and total hexosaminidase were significantly decreased. The fundus examination showed cherry red spots at the macula.

**Conclusions:**

IESS can be an epileptic phenotype of infantile SD. Clinical phenotypes should be adequately collected in genetic testing. In the case of negative sequencing results, gene variant reanalysis can be performed when the patients show clinically suspicious indications.

## Background

Infantile Sandhoff disease (SD; OMIM 268800) usually develops within the first 6 months of life, mostly with psychomotor retardation and regression as the first symptom. The characteristic clinical symptoms include seizures, excessive startle responses, hypotonia, cherry red spots on the fundus of the eye, and optic atrophy. Usually, the disease has a poor prognosis among children, with death occurring at two to three years of age [1]. The *HEXB* gene is the only gene known to cause SD [2]. It is located on chromosome 5q13.3 and contains 14 exons distributed in approximately 40 kb of DNA [1], encoding a β-subunit containing 556 amino acids. Mutations of the *HEXB* gene can cause deficiencies of β-hexosaminidase A (HexA) and B (HexB) enzymes, leading to abnormal degradation of GM2 ganglioside and resulting in disease [1].

Infantile epilepsy spasm syndrome (IESS) is a special developmental epileptic encephalopathy in infancy, which is characterized by clusters of epileptic spasms. Most IESS patients show quite abnormal interictal EEG features with hypsarrhythmia or multifocal discharges [3]. Convulsive seizures are often accompanied by slow development, arrest or regression.

Thus far, there have been no reports of infantile SD with onset of IESS. Here, we report a case of infantile SD with IESS as the predominant epileptic phenotype. This case was finally diagnosed with SD by reanalysis of the genetic raw data due to the remarkable clinical phenotype.

## Case presentation

### Clinical course

**Fig. 1:**
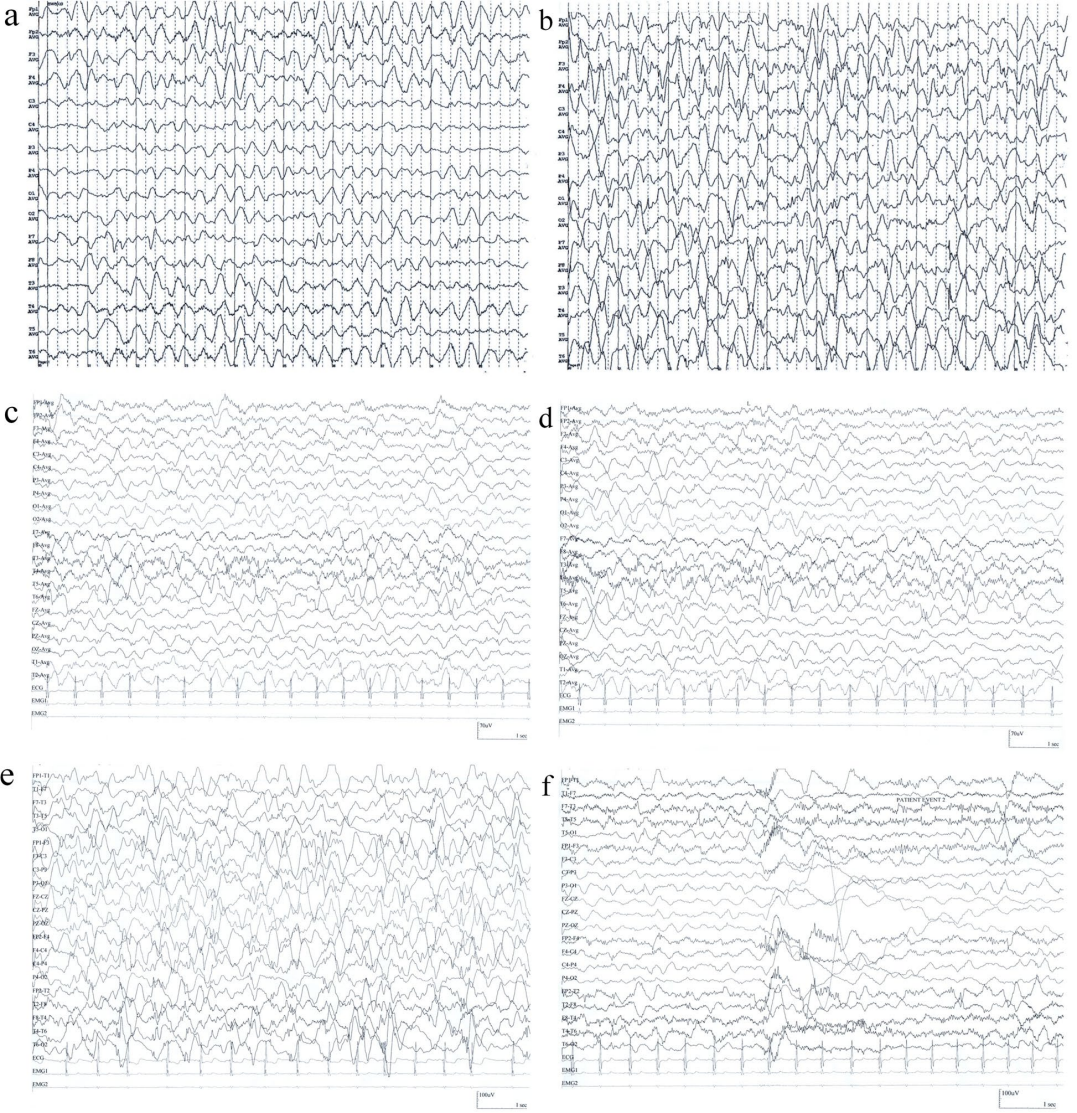
EEG recordings of the case. **a** The background EEG at the age of 16 months. **b** EEG recordings at the attack period of at the age of 16 months. **c** The background EEG at the age of 22 months. **d** The EEG recordings during a tonic seizure at the age of 22 months. **e** Atypical high arrhythmia during sleep at the age of 22 months. **f** The EEG recordings during a sound-evoked startle response in the child, manifested as eye squeezing, nervousness, and shoulder lifting

The case was a 22-month-old boy who was born at 38^+1^ weeks with a birth weight of 2.7 kg. He was born to healthy nonconsanguineous parents as a second child, with no significant family history of disease. At 6 months old, the child still could not sit steadily by himself, and rehabilitation training did not have any effect. At 11 months old, he could not roll over, sit alone, or grasp objects, and had poor eye-tracking and sound-tracking ability. The Peabody Motor Development Scale test showed that the child had approximately 4-month delay of motor development. After a respiratory infection at 13 months of age, the child showed obvious regression of psychomotor development and hypotonia. At the age of 16 months, he had seizures that manifested as nodding, lifting of both upper extremities, and clusters of epileptic spasms (Fig. 1a, b). At the age of 18 months, he had a tonic seizure with generalized rigidity and extension of both upper limbs, with head tilting back, eyes rolling upward, and loss of consciousness. The seizure was self-resolving and lasted for approximately 5 s. The frequency was a single attack every 1–2 weeks. Upon sound stimulation, the patient showed a startle response with postural changes. Video-EEG showed paroxysmal delta activities during wakefulness with multifocal epileptiform discharges significant in bilateral frontal regions, and a high degree of arrhythmia during sleep (Fig. 1c–f). Cranial MRI showed abnormal signals in the bilateral basal ganglia and periventricular white matter and a thin corpus callosum. The patient was diagnosed with infantile epileptic spasm syndrome. Topiramate, prednisolone acetate, clobazam and vigabatrin were added successively, but all showed poor efficacy.

**Fig. 2:**
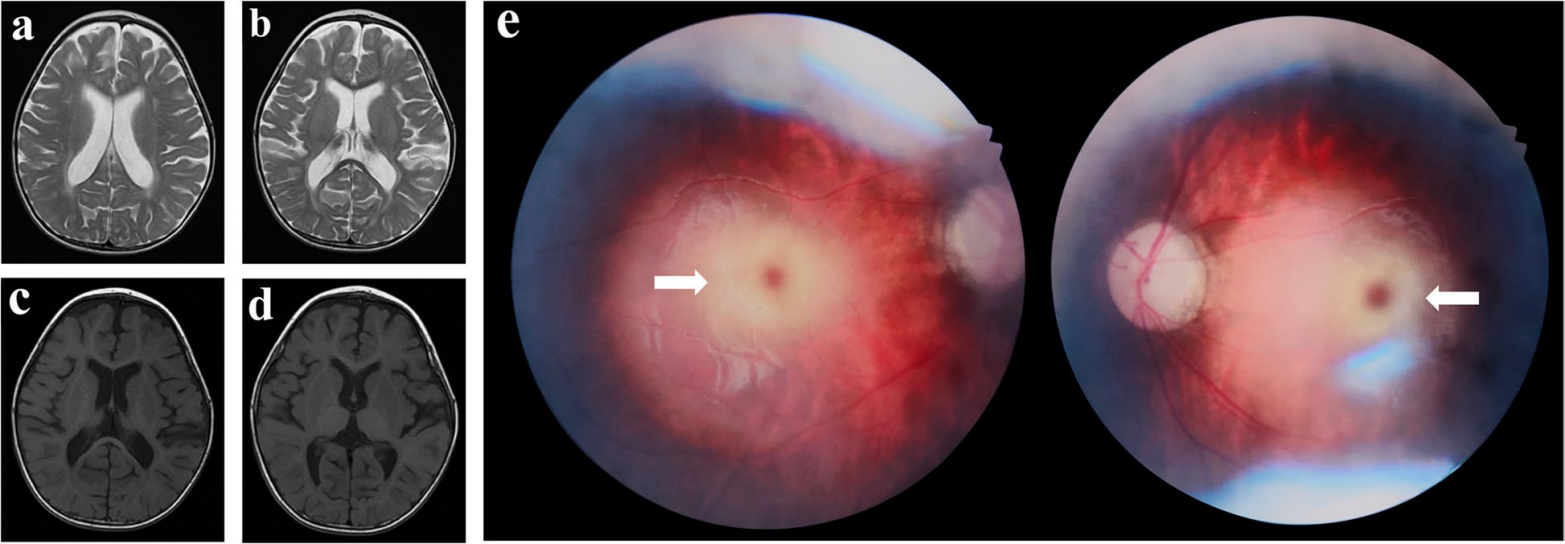
Cranial MRI of the case at age 22 months. **a**, **b** T2 weighted images; **c**, **d** T1 weighted images. Multiple symmetrical patches of slightly longer T1 and slightly longer T2 signals were seen in the white matter of the bilateral cerebral hemispheres and bilateral basal ganglia nuclei. Myelination of white matter is delayed. The corpus callosum is slender. The ventricular system, sulcus, fissure and pool are widened. **e** Fundus film of the case. The borders of the optic discs of both eyes were clear and pale, and the vascular pathways were acceptable, with cherry red spots visible in the macula. (white arrows)

The patient was admitted to our hospital at the age of 22 months. Physical examination indicated that he was flaccid, lethargic and severely malnourished. The patient had a head circumference of 48.2 cm. The liver could be touched at 1 cm below the costal margin of the midline of the right clavicle, and the spleen was not palpable. The body weight was below the 3rd percentile of other boys of the same age. No autonomous movement or sound chasing was observed. His bilateral pupils were equal in size, round and sensitive to light reflection. He showed muscle hypotonia with a grade I strength. His breathing rate was 50 times/min, and thick breath sounds were present in both lungs with laryngeal stridor and coarse wet rales; phlegm in the throat was obvious after eating. Sudden sound stimulation could induce tonic seizures. Video-EEG showed slow waves in the bilateral frontal, parietal, occipital, and temporal lobes, mixed with multifocal sharp waves and spikes, slightly significant in bilateral frontal and temporal regions. Cranial MRI showed multiple abnormal brain signals and sulcus fissure widening (Fig. 2a–d). Prednisone acetate and aminoglutethimide were gradually discontinued, and sodium valproate and levetiracetam were added for anti-seizure treatment, but the efficacy was poor.

### Reanalysis of raw data of genetic sequencing for genetic variation

**Fig. 3:**
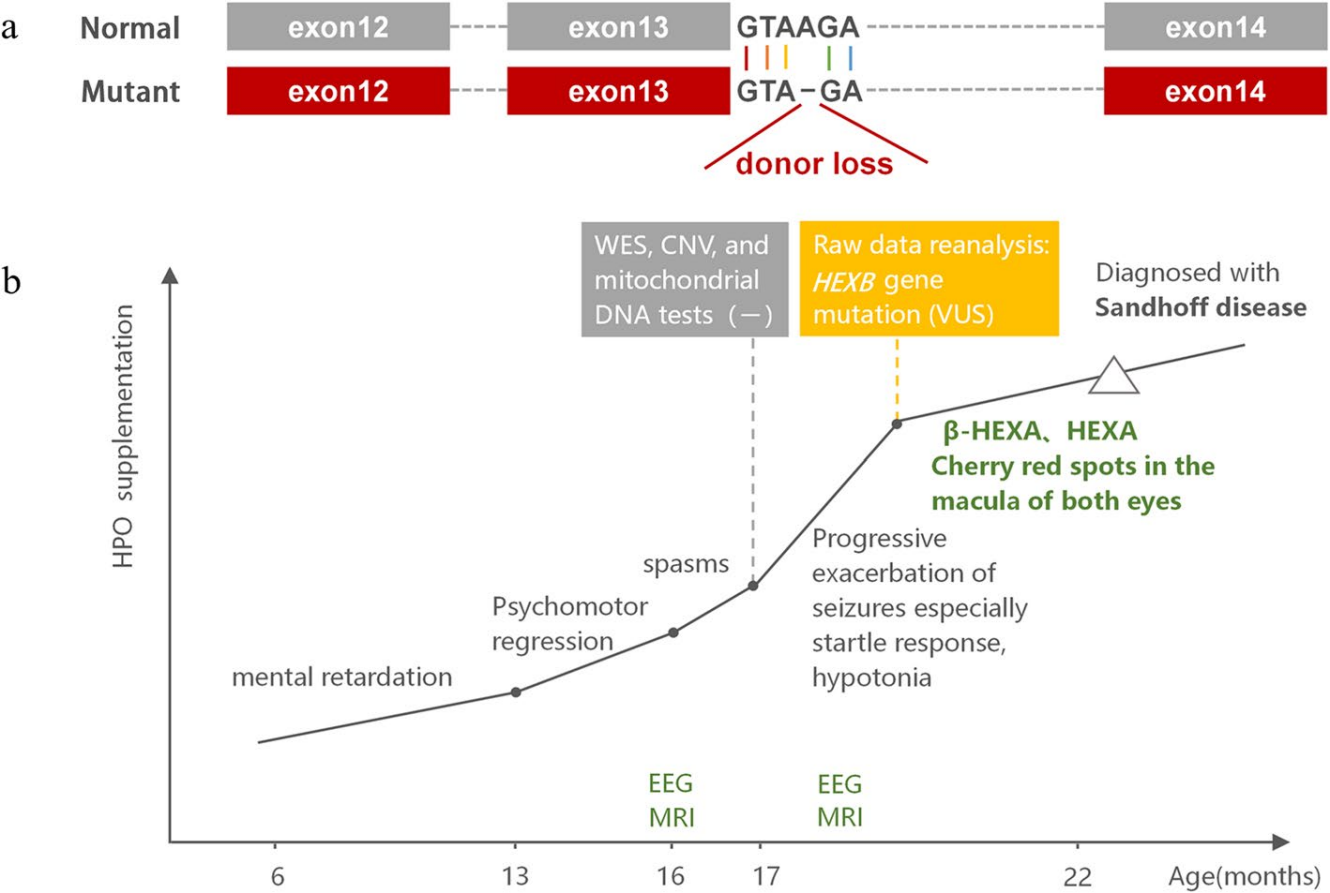
**a** The mutation site of the *HEXB* gene. Donor loss indicates the missing site. **b** The diagnostic history of the patient. WES: whole-exome sequencing; CNV: copy number variants; VUS: variants of uncertain significance

The whole exome sequencing (WES), copy number variant and mitochondrial DNA test data of all three members of the family obtained from the hospital of previous visits all showed negative results. Bioinformatics analysis was repeated on the raw data obtained, and after quality control, the clean reads were mapped to the UCSC hg19 human reference genome using the Sentieon BWA software. The parameter BWA of the Sentieon software was used to remove the duplicated reads and correct the base so that the mapped reads could be used for detecting variations. SNP and InDel variants were detected by the parameter driver. Then, the data were transformed into the VCF format. Variants were further annotated by the ANNOVAR software and associated with multiple databases, such as 1000 Genomes, ESP6500, dbSNP, EXAC, based on the inheritance patterns, variant types, population frequencies, and lists of genes associated with the main phenotypic characteristics of patient variants. Variant pathogenicity was predicted with the REVEL, SIFT, PolyPhen-2, MutationTaster and GERP +  + softwares. Possible disease-associated variants were confirmed by Sanger sequencing. The reanalysis of the original data revealed that the child had a homozygous mutation at the c.1613 + 4del splicing site of the *HEXB* gene (Fig. 3a), and both parents had heterozygous mutations.

### Complementary examinations, treatment and prognosis

Enzymatic activity assessment indicated that the activity of HexA was 16.6 nmol/g per minute (54.5–140.3) and that of the total hexosaminidase was 2.7 nmol/mg per hour (75.4–158.6), both significantly decreased. Ophthalmological examination of the fundus film showed a cherry red spot in the central fovea of each eye (Fig. 2e).

Combined with the clinical manifestations, enzymatic activity results and other auxiliary examinations, the patient was finally diagnosed with infantile SD (Fig. 3b). After oral administration of levocarnitine, vigabatrin, sodium valproate, levetiracetam and clobazam for 2 months, his seizure frequency was slightly reduced, swallowing improved, and weight increased.

## Discussion

SD has been widely reported as a metabolic genetic disorder in the Jewish population in the United States s, but only a few cases have been reported in the Chinese population [4]. We search the Pubmed, MEDLINE and other databases and summarize the clinical manifestations of infantile SD with seizures reported in recent 20 years. The main forms of seizure in infantile SD are generalized tonic–clonic and myoclonus seizures (Table 1). Other common manifestations are exaggerated startle responses to noise, psychomotor developmental delay/regression and presence of macular cherry red spot. This boy experienced tonic, tonic–clonic and myoclonic seizures successively, which is consistent with the literature. However, IESS was the initial epilepsy phenotype, which had never been reported in the literature. This case expands the seizure phenotype of infantile SD.

**Table 1:** Summary of common clinical manifestations in 27 cases of infantile SD

Patient No. and reference	Gend-er	Onset age	Diagnosis age	Form of seizure onset	Other clinical manifestations						Prognosis
					Exaggerated startle response to noise	Psychomotor developmental delay/ regression	Macular cherry red spot	Enlarged liver and spleen	Macroce-phaly	Other findings	
1 [1]	M	6m	17m	GTCS, MCS	+	+	+	-	-	MVP, hyperreflexia	Died at 2y
2 [1]	M	8m	25m	MCS	+	+	+	-	-	Hyperreflexia	Died at 4y
3 [1]	F	5m	16m	GTCS	+	+	+	Mild hepatomegaly	-	Axial hypotonia, Hyperreflexia	Died at 2y
4 [1]	F	8m	34m	MCS	+	+	+	-	-	Hyperreflexia	Died at 4y
5 [5]	M	6m	12m	Right-sided FS	+	+	+	-	-	Decreased vision	NA
6 [6]	M	6m	16m	GTCS	+	+	+	-	-	Hypotonia, brisk muscle stretch reflexes, a positive bilateral Babinski’s sign, microcephaly	Succumbed to an intercurrent respiratory illness at 18m
7 [7]	M	8m	22m	MCS, GTCS	+	+	+	+	-	Axial hypotonia with muscle weakness and brisk reflexes	NA
8 [8]	F	6m	15m	Recurrent seizures	-	+	+	-	-	Hypertonia, brisk tendon reflexes	NA
9 [8]	F	10m	12m	Recurrent seizures	-	-	-	-	-	Hypertonia, brisk tendon reflexes	NA
10 [8]	M	1m	8m	Recurrent seizures	+	+	-	-	-	Hypotonia, progressive muscular weakness, loss of motor skills	NA
11 [8]	F	8m	15m	Recurrent seizures	-	-	+	-	-	Hypertonia, brisk tendon reflexes	NA
12 [9]	M	6m	14m	GTCS, MCS	-	+	+	-	+	Poor sense of orientation, partly dilated of pupils, reacted sluggishly	Progressive neurologic deterioration with intercurrent respiratory infections
13 [10]	M	6m	18m	Myoclonus of the face and upper limbs	-	+	+	-	+	Axial hypotonia	NA
14 [11]	M12/F13	6.4m	14m	Lower limbs spasticity (56%)	-	+ 23 patients	+ 17 patients	+ 2 patients	-	Hypotonia, poor fix and follow and abnormal eye movement	11 patients died due to aspiration pneumonia and intractable seizure
20 [4]	F	6m	14m	Recurrent seizures	-	+	+	-	-	MR, hyperreflexia	NA
21 [4]	F	4m	8m	Recurrent seizures	+	+	+	-	-	Hyperreflexia	NA
22 [12]	F	2w	3y6m	FS	+	+	+	-	+	Ventricular septal defect, central hypotonia, peripheral hypertonia, a positive Babinski’s sign	Died at 3y6m
23 [13]	F	6m	22m	GTCS	-	+	+	+	+	-	NA
24 [14]	M	10m	10m	GTCS	-	+	+	-	-	Hypotonia, microcephaly, contracture in the bilateral metacarpophalangeal joint	NA
25 [14]	F	1y	1y6m	GTCS	-	+	+	Hepatomegaly	-	café-au-laid spots, hypotonia with brisk reflex	NA
26 [14]	M	5m	11m	GTCS	+	-	+	-	-	CDM	NA
27 [14]	F	1y3m	1y3m	FS	-	+	+	-	+	CDM	Died at 1y9m

*Abbreviations*: *M* male, *F* female, *y* years, *m* months, *GTCS* generalized tonic–clonic seizures, *MCS* myoclonic seizures, *FS* focal seizures, *MR* mitral regurgitation, *MVP* mitral valve prolapse and regurgitation, *CDM* congenital dermal melanocytosis, *NA* not available

The Human Phenotype Ontology (HPO) is a comprehensive resource for systematically defining and integrating human phenotypes and contributes to prediction of pathogenic genes. Studies have demonstrated the importance of deep phenotyping in improving the diagnostic power of clinical exome sequencing [15]. The phenotype-centric and phenotype-driven selection of genes can avoid missed diagnoses [16]. In this case, mental retardation and poor myelination were initially sent as HPO for testing, but the results were negative, which means no pathogenic gene consistent with clinical manifestations was found. Reanalysis of the original data after adding epilepsy, excessive startle response, and hypotonia to the HPO revealed a genetic variant that was consistent with the clinical phenotype. We speculate that the inadequate combination of the data with the clinical manifestations may have resulted in inaccurate phenotype entry, or that practitioners may have only focused on the positions of canonical “ ± 1–2” during analyzing, ignoring the possible impact of “ + 4” on splicing, resulting in the failure to detect any variant during the initial genetic testing of the case. The homozygous mutation of the c.1613 + 4del splice site of the HEXB gene in this child has not been previously reported. Our result extends the genetic spectrum of infantile SD.

## Conclusions

IESS can be the epileptic phenotype of infantile SD. Genetic testing and enzymatic activity testing are helpful in obtaining a definitive diagnosis. Genetic testing results should be comprehensively analyzed in combination with clinical evaluations. Genetic factors cannot be ruled out for difficult-to-interpret clinical manifestations in the nervous system. If necessary, the original genetic data can be reanalyzed to provide supportive evidence for disease diagnosis.

## Data Availability

The data that support the findings of this study are available on request from the corresponding author.

## References

[1] Tim-Aroon T, Wichajarn K, Katanyuwong K, Tanpaiboon P, Vatanavicharn N, Sakpichaisakul K, et al. Infantile onset Sandhoff disease: clinical manifestation and a novel common mutation in Thai patients. BMC Pediatr. 2021;21(1):22. 33407268 10.1186/s12887-020-02481-3 PMC7789739

[2] Stenson PD, Mort M, Ball EV, Evans K, Hayden M, Heywood S, et al. The Human Gene Mutation Database: towards a comprehensive repository of inherited mutation data for medical research, genetic diagnosis and next-generation sequencing studies. Hum Genet. 2017;136(6):665–77. 28349240 10.1007/s00439-017-1779-6 PMC5429360

[3] Zuberi SM, Wirrell E, Yozawitz E, Wilmshurst JM, Specchio N, Riney K, et al. ILAE classification and definition of epilepsy syndromes with onset in neonates and infants: Position statement by the ILAE Task Force on Nosology and Definitions. Epilepsia. 2022;63(6):1349–97. 35503712 10.1111/epi.17239

[4] Liu M, Huang D, Wang H, Zhao L, Wang Q, Chen X. Clinical and Molecular Characteristics of Two Chinese Children with Infantile Sandhoff Disease and Review of the Literature. J Mol Neurosci. 2020;70(4):481–7. 31919734 10.1007/s12031-019-01409-6

[5] Gowda VK, Amoghimath R, Srinivasan VM, Bhat M. Sandhoff Disease without Hepatosplenomegaly Due to Hexosaminidase B Gene Mutation. J Pediatr Neurosci. 2017;12(1):78–9. 28553389 10.4103/1817-1745.205623 PMC5437798

[6] Maulik K, Kumar S, Singh P, Saini AG. Microcephaly in infantile Sandhoff's disease. BMJ Case Rep. 2017;2017:bcr2017220912. 10.1136/bcr-2017-220912 PMC5535109 28647713

[7] Muralidharan CG, Tomar RPS. Infantile Sandhoff Disease: Unusual presentation. Med J Armed Forces India. 2016;72(Suppl 1):S91–3. 28050081 10.1016/j.mjafi.2015.11.008 PMC5192172

[8] Zhang W, Zeng H, Huang Y, Xie T, Zheng J, Zhao X, et al. Clinical, biochemical and molecular analysis of five Chinese patients with Sandhoff disease. Metab Brain Dis. 2016;31(4):861–7. 27021291 10.1007/s11011-016-9819-9

[9] Jain A, Kohli A, Sachan D. Infantile Sandhoff’s disease with peripheral neuropathy. Pediatr Neurol. 2010;42(6):459–61. 20472204 10.1016/j.pediatrneurol.2010.02.007

[10] Saouab R, Mahi M, Abilkacem R, Boumdin H, Chaouir S, Agader O, et al. A case report of Sandhoff disease. Clin Neuroradiol. 2011;21(2):83–5. 21153386 10.1007/s00062-010-0035-4 PMC3145082

[11] Tavasoli AR, Parvaneh N, Ashrafi MR, Rezaei Z, Zschocke J, Rostami P. Clinical presentation and outcome in infantile Sandhoff disease: a case series of 25 patients from Iranian neurometabolic bioregistry with five novel mutations. Orphanet J Rare Dis. 2018;13(1):130. 30075786 10.1186/s13023-018-0876-5 PMC6091055

[12] Sahyouni JK, Odeh LBM, Mulla F, Junaid S, Kar SS. Al Boot Almarri NMJ Infantile Sandhoff disease with ventricular septal defect a case report. J Med Case Rep. 2022;16(1):317. 36002893 10.1186/s13256-022-03550-0 PMC9404584

[13] Beker-Acay M, Elmas M, Koken R, Unlu E, Bukulmez A. Infantile Type Sandhoff Disease with Striking Brain MRI Findings and a Novel Mutation. Pol J Radiol. 2016;81:86–9. 26985245 10.12659/PJR.895911 PMC4780271

[14] Ozaal S, Jayasena S, Jayakody S, Schröder S, Jayawardana A, Jasinge E. Clinical Presentation and Genetic Heterogeneity Including Two Novel Variants in Sri Lankan Patients With Infantile Sandhoff Disease. Child Neurol Open. 2022;9:2329048X221139495. 36407556 10.1177/2329048X221139495 PMC9673506

[15] Robinson PN. Deep phenotyping for precision medicine. Hum Mutat. 2012;33(5):777–80. 22504886 10.1002/humu.22080

[16] Tomar S, Sethi R, Lai PS. Specific phenotype semantics facilitate gene prioritization in clinical exome sequencing. Eur J Hum Genet. 2019;27(9):1389–97. 31053788 10.1038/s41431-019-0412-7 PMC6777628

